# A case of retractable helix lead auto‐retraction: A possible cause of deep septal lead dislodgement

**DOI:** 10.1002/joa3.12891

**Published:** 2023-07-08

**Authors:** Keisuke Suzuki, Eiji Sato, Yoshihiro Yamashina, Akihiko Ishida, Tetsuo Yagi

**Affiliations:** ^1^ Department of Cardiovascular Medicine Sendai City Hospital Sendai Japan

**Keywords:** complication, deep septal pacing, lead dislodge, pacemaker, self‐retracted lead

## Abstract

This paper explains the phenomenon where the helix lead automatically retracts because of residual torque during deep septal pacing.

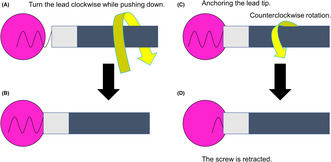

A woman in her 70s was admitted to our hospital with symptoms of syncope and sick sinus syndrome (SSS) that necessitated pacemaker implantation. The preoperative 12‐lead electrocardiogram showed sinus rhythm without conduction disturbances (Figure 1A). The patient had a history of both aortic valve replacement and breast cancer.

**FIGURE 1:**
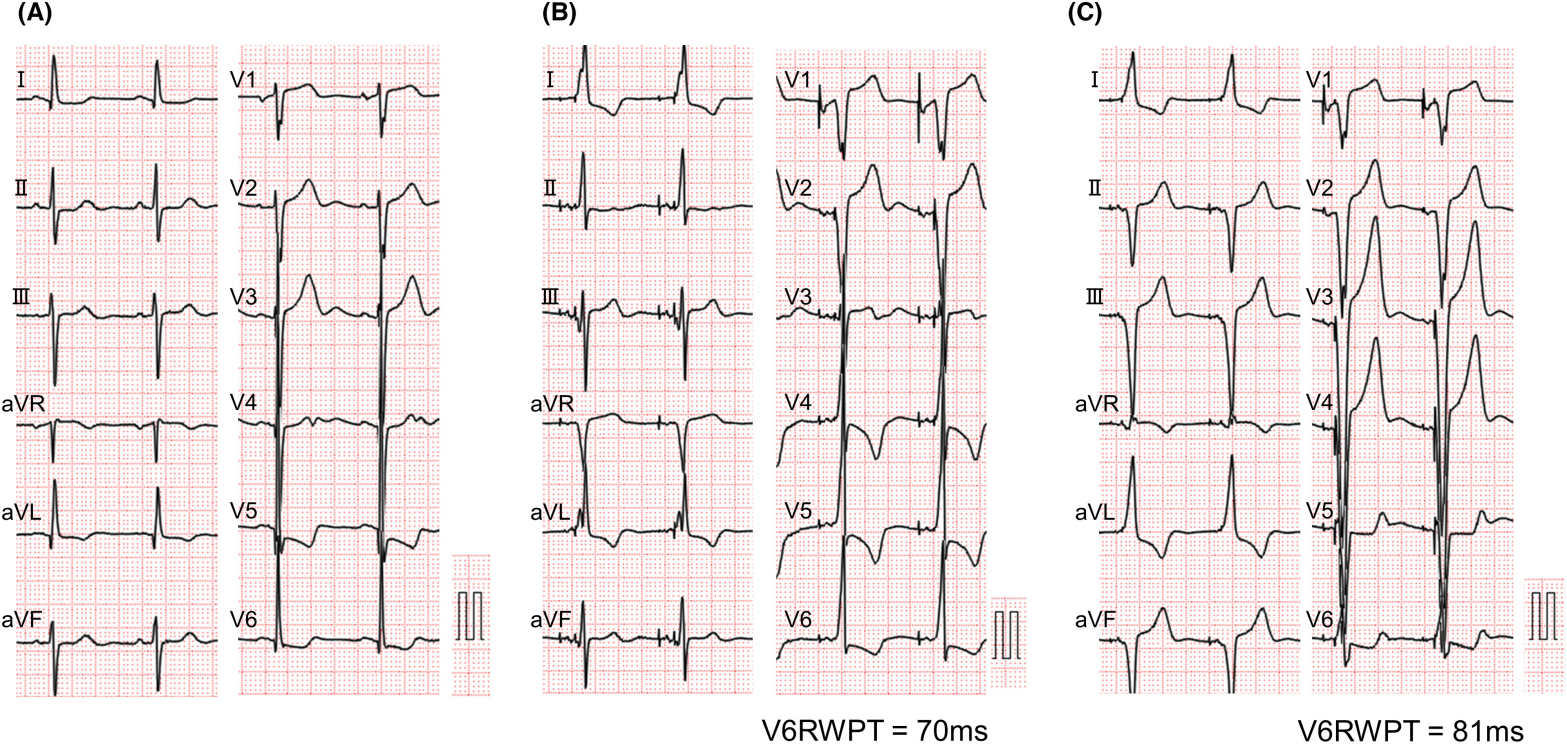
Comparison of the 12‐lead electrocardiogram before and after implantation. (A). The 12‐lead electrocardiogram before the implantation of the pacemaker showed sinus rhythm with a heart rate of 67 bpm. (B) The 12‐lead electrocardiogram taken on the first postoperative day showed atrial and ventricular pacing with a heart rate of 70 bpm. V6RWPT was 70 ms. (C) The 12‐lead electrocardiogram taken after inferior septal pacing showed atrial and ventricular pacing with a heart rate of 70 bpm. V6RWPT was 81 ms. RWPT, R‐wave peak time.

As a result of her prior history of breast cancer and lymph node dissection on the left side, pacemaker implantation was performed from the right side. A screw lead was placed in the right basal septum using the guiding sheath (Selectra 3D‐55‐39; BIOTRONIK, Berlin, Germany) to perform pacing in the right ventricular septal area (Figure 2A). The screw‐in lead (Solia S 60; BIOTRONIK) was rotated clockwise while being pressed against the myocardium after turning the connector pin. When rotating the lead itself, we made four turns while using a green attachment on the connector pin as previously reported.^1^ On the postoperative day, the 12‐lead ECG showed R waves were missing on the V1 lead but the duration of R‐wave peak time on the V6 lead was shortened to 70 ms, which was indicative of deep septal pacing (Figure 1B).^2^ Two days after the surgery, a change in the pacing waveform was observed and subsequent X‐ray showed that the lead position was displaced as compared to the position on the previous day (Figure 3A,B). X‐ray imaging revealed a retraction of the screw from its previous position (Figure 2D,F). After confirming that the screw was functioning properly, re‐implantation of the pacemaker into the lower septum was performed (Figure 3C), and the patient was discharged without any complications.

**FIGURE 2:**
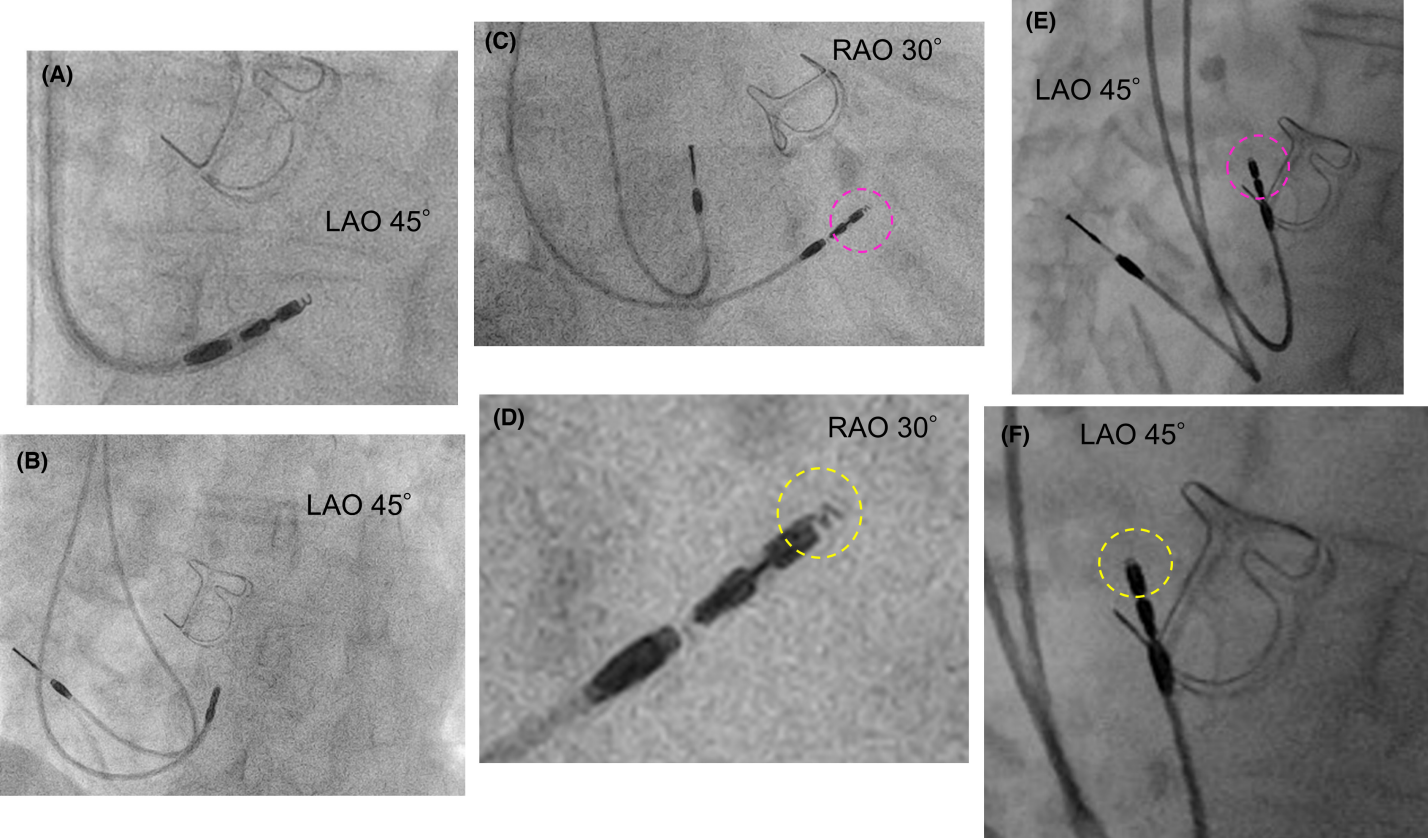
Comparison of screw position before and after lead dislodgement. (A) The lead was positioned in the right ventricular basal septum using a guiding sheath. Afterward, the lead was rotated clockwise (LAO 45°). (B) Position of the leads after slitting out the guiding sheath after deep septal pacing (LAO 45°). (C) The pink dotted circle represents the tip of the ventricular lead after deep septal pacing (RAO 30°). (D) The enlarged picture of the ventricular lead is shown in Figure 2C. The yellow dotted circle shows that the screw extended appropriately from the lead. (E) The pink dotted circle represents the tip of the ventricular lead after dislodgement (LAO 45°). (F) The enlarged picture of the ventricular lead is shown in Figure 2E. The yellow dotted circle represents that only one turn of the screw was protruding from the lead tip. Checking from multiple angles, the screw most protruded at this angle. LAO, left anterior oblique; RAO, right anterior oblique.

**FIGURE 3:**
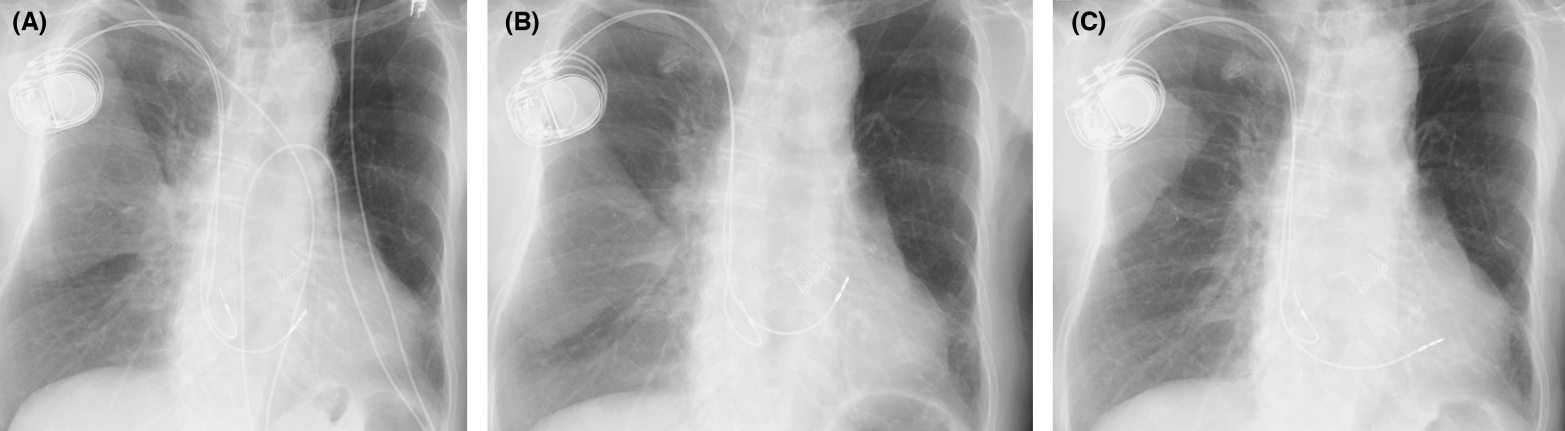
Chest X‐ray images comparing lead positions. (A) The anteroposterior chest X‐ray on the first postoperative day revealed the placement of a ventricular lead at the basal right ventricular septum. (B) The anteroposterior chest X‐ray image was taken after the lead dislodgement on the second postoperative day. The ventricular lead shifted superiorly and anteriorly compared to its position on the previous day. (C) The anteroposterior chest X‐ray image was taken after inferior septal pacing.

In this particular case, deep septal pacing was performed as previously reported.^1^ The tip of the screw protruded adequately during the first implantation, but it was found to have retracted when the lead was dislodged. Although we cannot rule out the possibility of a micro‐dislodge to a complete dislodge, an experiment was conducted to investigate the phenomenon. When the lead was rotated counterclockwise while holding the tip of the lead onto the myocardium, the screw was shown to be pulling back (Supplemental Video S1). If there were any residual torque on the lead while its tip is secured to the myocardium, it can lead to a gradual counterclockwise rotation of the lead, ultimately resulting in the screw being pulled back (Figure 4), which may lead to changes in lead parameters and eventually result in lead dislodgement. This phenomenon can potentially be prevented by applying additional torque to the lead to counteract the returning torque or by manipulating the lead with a stylet to remove any torque building up after fixation. Attention should be paid to the remaining torque when turning the screw‐in lead itself during deep septal pacing.

**FIGURE 4:**
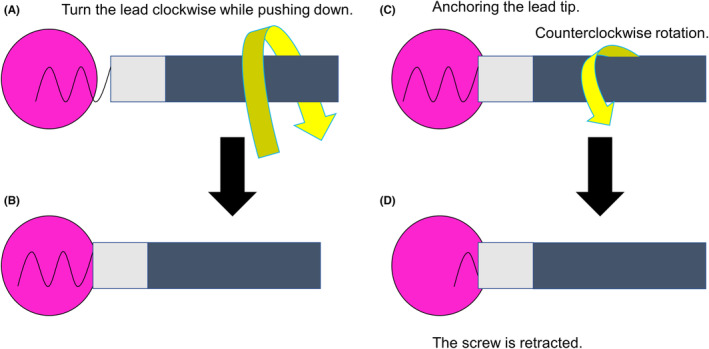
Schematic of deep septal pacing using a screw‐in lead and the phenomenon of auto lead retraction. (A) While the screw is inserted into the myocardium, the lead body is rotated in a clockwise direction while being pushed down. (B) The screw can be implanted deeply into the myocardium by rotating the lead body clockwise. (C) The lead body is rotated counterclockwise while the tip of the lead remains anchored. (D) The screw is stored inside the lead.

## FUNDING INFORMATION

None.

## CONFLICT OF INTEREST STATEMENT

The authors declare that they have no conflicts of interest regarding this work.

## ETHICS STATEMENT

The research related to human use has complied with all the relevant national regulations and institutional policies, as well as is in accordance with the tenets of the Helsinki Declaration.

## PATIENT CONSENT STATEMENT

Informed consent was obtained from the patient featured in this case report for inclusion in the publication. The patient was fully informed about the purpose, risks, as well as benefits of this report, and has given her voluntary agreement to participate. I confirm that the patient's privacy will be protected, and the patient's personal information will remain confidential.

## PERMISSION TO REPRODUCE MATERIAL FROM OTHER SOURCES

None.

## Supporting information


Supplemental Video S1.

## Data Availability

The datasets generated and/or analyzed during the current study are available from the corresponding author upon reasonable request.
